# The Vital Action of the Dental Pulp

**Published:** 1904-10

**Authors:** R. R. Andrews

**Affiliations:** Cambridge, Mass.


### THE VITAL ACTION OF THE DENTAL PULP.[1]

BY R. R. ANDREWS, A.M., D.D.S., CAMBRIDGE, MASS.

I HAD the honor, several years ago, of reading a paper before this Section on "The Embryology of the Dental Pulp." This paper gave a minute description of the various processes taking place during the development of the tooth from its formative pulp. I propose in the present essay to consider the nature of the mature pulp, and to call your attention to its vital action after the tooth is formed. I shall consider the pulp within the pulp-chamber and its myriad fibrils, for these fibrils are as much a part of the pulp as any portion of it, and are the channels by which its vital functions are carried on. These canals are slightly undulating, and radiate from the pulp-chamber to the outer surface of the dentine. Each canal contains a fibre bathed in a fluid, and this fibre is an arm of the pulp. Branches from this fibre anastomose with others through the dentine matrix. They form a delicate net-work in the substance of the crown near the enamel.

In the region of the cementum they anastomose with the fibres of the granular layer of the root. When the tooth is fully formed, the principal function of the pulp is for the vitalization of the substance of the dentine by means of its fibrils, which permeate into every portion of the matrix of the dentine. Its function is not only to vitalize, but it may again assume its formative function whenever causes for repair demand this action.

One of the difficulties we find in our research work on the mature pulp is the fact that we can not look on its tissue in life. We can not see these vital processes while they are going on, but must make our deductions on freshly extracted normal teeth and pulps that are as near the life period as possible. But it is always dead tissue that we have to examine. We draw our conclusions from what is shown to have taken place when the tissue was alive; we know that the living pulp, with its blood-vessels and nerves, nourishes the dentine; that vital changes do take place, and that this pulp is the source of vital action. It is a living organ, subject

---

[1] Read at the annual session of the American Medical Association, Section on Stomatology, Atlantic City, June 7 to 10, 1904.

to any physiologic or pathologic process, which may act on any living matter; therefore, we may expect to find its connection with the general economy similar to that of other tissues. It will respond to the action of returning health, and caries which have commenced have been arrested by this vital action. They appear as polished blotches on the teeth and are not uncommon. Professor miller, in his work on "Micro-Organisms of the Human Mouth," calls this condition a spontaneous healing of dental decay. The dentine, which had become softened, has become hard again, and the decaying process is stopped. This change also takes place in the temporary teeth. The healed dentine retains its discolored appearance, but becomes nearly as dense as normal dentine. These changes have been brought about by vital action, and this action came from the agency of the pulp.

The histologic structure of the normal pulp, at the time of the full formation of the tooth, as has already been described in a former paper, is as follows: At the periphery we have the pear-shaped cells, then the spindle-shaped conjugation layer of cells, then the spindle-shaped and irregularly shaped cells with their anastomosing processes, and lastly, the connective tissue elements in the central portion of the pulp, which seem to be scant in protoplasm. These cells are not very numerous, and are in a jelly-like matrix. The blood-vessels enter at the apex, the trunk-vessels resting near the centre of the pulp. Sometimes as many as three arteries are seen to enter the apical foramen. They then divide into innumerable branches and form an extensive net-work of capillaries near the layer of the pear-shaped cells next the formed dentine. There are numerous veins also found, but these are somewhat larger than the arteries. Black tells us that the blood-vessels of the pulp are remarkable for the thinness of their walls, and that the smaller veins seem to be nothing more than endothelial cells which are placed edge on edge, or margin on margin. The arteries have a circular and longitudinal layer of muscular fibres, but these are very thinly distributed.

There is always an effort on the part of the pulp to protect the dentine from destruction from whatever cause. A microscopic examination shows us how misleading it is to call this organ a nerve. Its matrix is a mass of connective tissue, in the substance of which we find nerve-fibres, medullated and non-medullated. These enter the pulp through the apical foramen in bundles of various sizes. As they pass into the pulp they break into branches and form a rich net-work, a delicate plexus of fine nerve-filaments, next the outer pear-shaped cells. It is not certainly known how they communicate with the fibril. It has been suggested that the finer fibres may pass between the pear-shaped cells, winding themselves around the dentinal fibrils and thus pass into the dentinal canal. There is also a rich capillary net-work of blood-vessels near these pear-shaped cells in the newly formed tooth, and when we inject these and examine them under the microscope there seems to be little room left for other tissues there. When the dentine is irritated by infection or its surface is uncovered by a break, there immediately follows a period of vital activity. If we examine sections of a tooth made when these changes are taking place, we shall see that the formative cells in that portion of the pulp nearest the point of repair are filling up with glistening globular bodies, and the tissue about it is showing an increased vascularity, as though an active formative action were taking place; and in the canals opening towards the area of irritation, within the dentine matrix, we find minute glistening granules, which are being carried outwardly towards the point of lesion. These glistening particles have the appearance of being minute calco-spherites. In studying this condition, some years ago, I satisfied myself that these appearances were the result of the vital action of the pulp in its efforts to repair the tissue, and that the minute glistening particles within the canals were in many ways similar to the minute globular bodies found in the tissues while the dentine matrix was developing. They are being forced into and through the canals of the matrix to the point of irritation, and I have seen long lines of them in the canals, nearly filling them up. In favorable cases the canals against the irritation do become filled and a formative process goes on within the pulp-chamber, until a calcified barrier is formed there, corresponding to the part disturbed or destroyed. When this change takes place the consolidated dentine in this area becomes slightly darker in color than normal tooth-structure, and might easily be mistaken for decay.

In carious pits and fissures of the bicuspids and molars the organisms of infection proceed inward through the dentinal canals towards the pulp. As it nears the pulp this protecting barrier is formed, and under normal conditions the infection is retarded, changes its course, and moves in the next weaker direction towards the approximal surface, usually without exposing the pulp—if taken in time. We also find the protecting consolidation in teeth that are worn down, usually in the mouth of old people, and when this change has taken place these teeth are not liable to decay again, except under very unfavorable circumstances. This protecting process forms that tissue known as the zone of resistance; the hyaline appearance of this zone tissue under the microscope is caused by the lime globules consolidating the canals that are in the substance of the zone. These changes are due, in a large measure, to normal conditions, as regards the vitality of the individual: But in cases where the constitutional condition is below the normal, even where they seem favorable to decay, there is always an attempt made to retard the infection. Under certain conditions of environment and infection, penetrating decay is so rapid that the vital action of the pulp is overwhelmed; and the pulp becomes exposed, and is in a pathologic condition even before the breaking away of the cavity walls.

The pulp is the central and largest source of vitality to the tooth, and it acts through its myriads of fibrils. Sometimes the ends of the fibrils are seen to be running slightly into the enamel substance. In the root portion they anastomose with the fibrils of the granular layer near the cement, and a communication is seen, in many cases, to be continued through the cementum by means of the lacuna and their fibrils, and in a few cases I have traced them out to the pericementum. Pain of the dentine, following the touch of an instrument, or from any irritation, is expressed through the agency of these fibrils, and we become conscious of the sensation through them. When irritation is caused by wear, erosion, or a break exposing the dentine, a section under the microscope will show that secondary dentine has been formed within the pulp-chamber, and this corresponds to the loss of substance of the dentine which has been affected. This secondary dentine is a tissue that has been called dentine of repair, and this is a manifestation of the vital action of the pulp. It is formed within the pulp-chamber, and is always an addition to the already formed dentine. It forms against the portion of the pulp-cavity next to the fibrils which have been affected by the lesion. The enamel might wear or break indefinitely, and we shall find no compensation of any kind occurring until it reaches the surface of the dentine, whereon the vital power of the pulp is aroused and an action of repair progresses in proportion to the extent of the injury. Some have thought these changes occur only in the teeth of old people, but such is not the case. They may occur at any age, and this process of repair has been found to have taken place in the tissues of a temporary tooth. These changes are all characteristic of the vital action of the pulp. The dentine is and was meant at all times to be a living tissue. As I have shown, it receives impressions of injuries and responds by processes of repair. Some of the ablest men in the profession have questioned the further value of the tooth-pulp after the full formation of the tooth has taken place. They look on it as simply a formative organ, and consider its mission closed with the formation of the tooth. It is, therefore, in their judgment quite as well to destroy it, take it out, and fill its chamber. The microscopic appearance of dentine, after the pulp is removed, shows that a large amount of dead organic tissue is left within the canals that cannot be taken out, and this dead tissue is a source of considerable danger to the health and vitality of the pericementum.

The subject of vitality, tissue repair, and compensation for injury on the part of the pulp should suggest a lesson for us all. The whole phenomena of vital action shows that the pulp is, under proper conditions, always helpful in bringing about successful results, if properly attended to. The restoration to a healthy condition of an irritated and troublesome pulp, is among the highest acts of professional skill. It is unfortunate that so many pulps have to be destroyed. It is fortunate that so many teeth remain quiet and apparently healthy after pulp extirpation and treatment. With the death of the pulp we lose not only sensation in the dentine, but also all the changes which vitality give to an organ, such as nutrition and recuperation. These can never by any possible means be revived. The main mass of the dentine of the tooth is dead. Myriads of lifeless fibrils are in its canals. It is true that the cementum, which was not formed from the pulp-tissue, does furnish a limited amount of vitality and nourishment to the root, which is covered by the pericementum; but the health of the pericementum is threatened by the dead tissue which is locked up in the canals within the dentine matrix. In vigorous health pulpless teeth have been successfully treated and remain serviceable for years. In cases of a lessened vitality, we may expect more or less pericemental trouble, a darkening of the tooth, a recession of the gums, and an absorbing of the alveolar processes. The tooth is beyond the influence of any systematic process, and there is no probability of a change for the better. Abscess and necrosis may supervene, and extraction is the last resort. I conclude by quoting an extract from a paper written in 1874 by Dr. J. E. Craven, who says,—

"Here is an organ formed of a delicate tissue, as is the eye, and because some agent of decay threatens its ivory walls, the ruthless hand of a blissful ignorance pours on its devoted head such destroying angels as carbolic acid, creosote, cobalt, and arsenous acid. Poor little pulp, you have been caught, and the destructive genius at the chair wills that you be deprived of your previous life. Why not lay aside those substances that blister and crisp the tissue until its life is enfeebled or lost, and, instead, resort to milder agents whose influence tends to cool the fevered part and allay the pain, reduce the inflammation, and use the food that nature herself would suggest to replace the covering the pulp has lost by decay?"

